# Probabilistic Solar Wind and Geomagnetic Forecasting Using an Analogue Ensemble or “Similar Day” Approach

**DOI:** 10.1007/s11207-017-1090-7

**Published:** 2017-04-19

**Authors:** M. J. Owens, P. Riley, T. S. Horbury

**Affiliations:** 1 Space and Atmospheric Electricity Group, Department of Meteorology, University of Reading, Earley Gate, PO Box 243, Reading, RG6 6BB UK; 2 Predictive Science Inc., 9990 Mesa Rim Rd, Suite 170, San Diego, CA 92121 USA; 3 Blackett Laboratory, Imperial College London, London, SW7 2BZ UK

**Keywords:** Solar wind, Space weather, Heliospheric magnetic field

## Abstract

Effective space-weather prediction and mitigation requires accurate forecasting of near-Earth solar-wind conditions. Numerical magnetohydrodynamic models of the solar wind, driven by remote solar observations, are gaining skill at forecasting the large-scale solar-wind features that give rise to near-Earth variations over days and weeks. There remains a need for accurate short-term (hours to days) solar-wind forecasts, however. In this study we investigate the analogue ensemble (AnEn), or “similar day”, approach that was developed for atmospheric weather forecasting. The central premise of the AnEn is that past variations that are analogous or similar to current conditions can be used to provide a good estimate of future variations. By considering an ensemble of past analogues, the AnEn forecast is inherently probabilistic and provides a measure of the forecast uncertainty. We show that forecasts of solar-wind speed can be improved by considering both speed and density when determining past analogues, whereas forecasts of the out-of-ecliptic magnetic field [$B_{\mathrm{N}}$] are improved by also considering the in-ecliptic magnetic-field components. In general, the best forecasts are found by considering only the previous 6 – 12 hours of observations. Using these parameters, the AnEn provides a valuable probabilistic forecast for solar-wind speed, density, and in-ecliptic magnetic field over lead times from a few hours to around four days. For $B_{\mathrm{N}}$, which is central to space-weather disturbance, the AnEn only provides a valuable forecast out to around six to seven hours. As the inherent predictability of this parameter is low, this is still likely a marked improvement over other forecast methods. We also investigate the use of the AnEn in forecasting geomagnetic indices Dst and Kp. The AnEn provides a valuable probabilistic forecast of both indices out to around four days. We outline a number of future improvements to AnEn forecasts of near-Earth solar-wind and geomagnetic conditions.

## Introduction

The purpose of space-weather forecasting is, ultimately, to improve decision-making capability for a range of end users, including (but not limited to) power companies, satellite operators, the aviation industry, communication companies, and human-spaceflight controllers (Hapgood 2011; Cannon *et al.* 2013). While accuracy is central to any useful forecast, different aspects of the forecast, *e.g.* ratio of false alarms to missed events, reliability of “all clear” forecasts, forecast lead time, correct event occurrence statistics, and ability to predict extremes, will be more or less important for different operational applications.

Physics-based, long lead-time (*i.e.* greater than approximately 40 minutes, the nominal $\mbox{L}_{1}$-to-Earth solar-wind propagation time) space-weather forecasting requires prediction of near-Earth solar-wind conditions on the basis of remote solar or heliospheric observations. This is typically provided by deterministic numerical magnetohydrodynamic (MHD) models (*e.g.* Riley, Linker, and Mikic 2001; Odstrcil *et al.* 2004; Tóth *et al.* 2005). Ensembles of numerical solar-wind MHD models, using multiple runs of the numerical model with stochastic perturbations to the initial conditions (*e.g.* Cash *et al.* 2015), are beginning to be used operationally, although the number of ensemble members is limited by computational power, and the range of perturbations is poorly constrained by observations. More fundamentally, it is not clear that the probability density function (PDF) generated from the spread in ensemble members accurately represents the uncertainty in the forecast. In particular, if the model has any systematic bias, such as an under-prediction of the heliospheric-magnetic-field (HMF) intensity [$B$], then the ensemble PDF of $B$ will also be skewed to low values and not represent the true likelihood of a given value of $B$.

Similar issues are faced by terrestrial atmospheric-weather forecasting. Before the advent of numerical weather prediction (NWP) models, and especially the forecast skill advance that came with the advent of ensemble NWP (*e.g.* Leutbecher and Palmer 2008 and references therein), “similar day” or analogue forecasting (AF) methods were widely used. The basic premise is that if close matches (or analogues) to the current atmospheric conditions can be identified in historical observations, these analogues will provide a good estimate of conditions in the future (*e.g.* van den Dool 1989 and references therein). By considering an “ensemble” of past analogues, a probabilistic forecast for future conditions can be constructed. As this is the result of observations, it is inherently bias-free. AF is actually of limited use for terrestrial weather forecasting as the atmospheric system is inherently chaotic, and so it is a poor assumption that two atmospheric states that are initially close will remain so in the future (Lorenz 1969). For solar-wind forecasting, which is far more of a system “driven” by boundary conditions, the prospects are more promising, as we shall demonstrate.

AF has since found a new role within atmospheric forecasting (Delle Monache *et al.* 2013). Historical NWP forecasts are analysed for times analogous to the current forecast state. These times are used to produce an ensemble of the observed conditions. This serves two purposes: Firstly, it corrects for any bias in the NWP forecast. Secondly, with little additional computational cost or need to specify initial condition perturbations, it transforms a single deterministic forecast into probabilistic forecast with an accurate bias-free assessment of the uncertainty in the forecast. This approach will undoubtedly be useful for solar-wind forecasting, once a long enough (*e.g.* decades) catalogue of historical MHD solar-wind forecasts has been amassed. At present, we investigate the use of a purely observation-based analogue ensemble (AnEn) for statistical solar-wind forecasting, rather than in conjunction with a model.

The concept of analogue or “similar day” forecasting has previously been investigated for specific space-weather uses, with pattern matching within discrete solar-wind events, specifically magnetic clouds (Chen, Cargill, and Palmadesso 1997) and high-speed streams (Bussy-Virat and Ridley 2016). More recently, Riley *et al.* (2017) have demonstrated the potential of analogue forecasts through simple pattern matching for various solar-wind conditions. Considering solar-wind parameters independently, and fixing the number of analogue periods at 50 and the period over which they are determined at 24 hours, they showed significant forecast skill for a few days lead time in solar-wind speed, density, and temperature, but only a few hours for the out-of-ecliptic magnetic-field component. Nonlinear approaches, particularly neural networks, which implicitly involve analogue-forecasting ideas, have been widely used for predicting Kp, one of the most widely used indices of geomagnetic disturbance (Detman and Joselyn 1999; Boberg, Wintoft, and Lundstedt 2000; Wing *et al.* 2005). These are typically used to assess the recent solar-wind conditions and give Kp forecasts with a lead time of about three hours (*e.g.* Solares *et al.* 2016). While not directly an analogue forecast as such, explicit decomposition of sunspot, irradiance, and geomagnetic time series into the frequency domain has also been shown to have predictive power over short (*e.g.* one- to seven-day) forecast lead times (Reikard 2016).

In this study, we build on the results of Riley *et al.* (2017) to investigate the purely AnEn approach for continuous probabilistic solar-wind and geomagnetic forecasting. We perform a sensitivity analysis of the AnEn forecast skill to the choice of the time period and parameters over which the analogy is computed, as well as the number of analogous periods used to produce the ensemble. We then quantify the potential economic value of the probabilistic AnEn forecasts relative to persistence and climatology.

## Producing an Ensemble Analogue (AnEn) for the Solar Wind

To produce a solar-wind AnEn forecast, we use the OMNI series of near-Earth *in-situ* spacecraft observations (King and Papitashvili 2005) at one-hour resolution. The inclination angle of the Ecliptic plane to the solar-rotation direction means that there are entirely geometric trends in the magnetic-field and solar-wind flow vectors. In geocentric-solar-ecliptic (GSE) coordinates, predictability is thus present in the $y$- and $z$-components of the solar-wind flow and magnetic-field time series that is unrelated to solar-wind structures or variability, but results purely from the variation in the GSE coordinate system over the Earth’s orbit (*e.g.* Rosenberg and Coleman 1969; Russell and McPherron 1973). In this study, data are considered in the heliographic radial-tangential-normal (RTN) coordinate system, where the normal is along the solar-rotation axis and the tangential is to the solar-rotation direction. The RTN coordinate system removes the orbital or geometric trends effects in the near-Earth solar-wind parameters and enables the predictability of solar-wind structures to be studied in isolation. For space-weather forecasting purposes, the radial solar-wind flow speed [$V_{\mathrm{R}}$] and the component of the HMF normal to the solar-rotation plane [$B_{\mathrm{N}}$] are the critical parameters, as they are the primary contributors to the dawn-to-dusk electric field that controls reconnection with the magnetospheric field (Dungey 1961). Owing to the inclination of the Earth’s magnetosphere and orbital plane to the solar-rotation plane, the HMF component along the solar-rotation direction [$B_{\mathrm{T}}$] also leads to a magnetic field anti-parallel to the nose of the magnetosphere, in a manner that varies systematically with both day of year and time of day (Lockwood *et al.* 2016). The solar-wind density [$N_{\mathrm{P}}$] also affects the compression of the magnetosphere and hence the efficiency of the magnetic coupling between the heliospheric and magnetospheric magnetic fields. These are the four solar-wind parameters investigated in this study.

Using OMNI data in RTN coordinates, the AnEn methodology is first demonstrated for the simplest case: forecasting a single solar-wind parameter [$P$] in near-Earth space from the current time [$t_{0}$] out to a forecast lead-time [$t_{0} + T_{\mathrm{F}}$], where $T_{\mathrm{F}}$ is the length of the forecast window. The similarity of recent solar-wind conditions to historic observations is quantified using the mean-square error (MSE) between $P$ during the training window, $t_{0} - T_{\mathrm{T}}$ to $t_{0}$ (where $T_{\mathrm{T}}$ is the length of the training window), and $P$ at all previously observed intervals, *i.e.* $t_{n} - T_{\mathrm{T}}$ to $t_{n}$, for all values of $t_{n}$ between the start of OMNI data and $t_{0}$. Obviously, for true forecasting purposes, only training intervals before $t_{0}$ will be available. For solar-wind AnEn development, however, the database can be enlarged by using intervals both before and after $t_{0}$, although the forecast window itself is obviously excluded. The analogues are the $N_{\mathrm{EN}}$ periods with the lowest MSE values. The forecast is then assembled from the ensemble of the $N_{\mathrm{EN}}$ time series over the period $t_{n}$ to $t_{n} + T_{\mathrm{F}}$.

**Figure 1:**
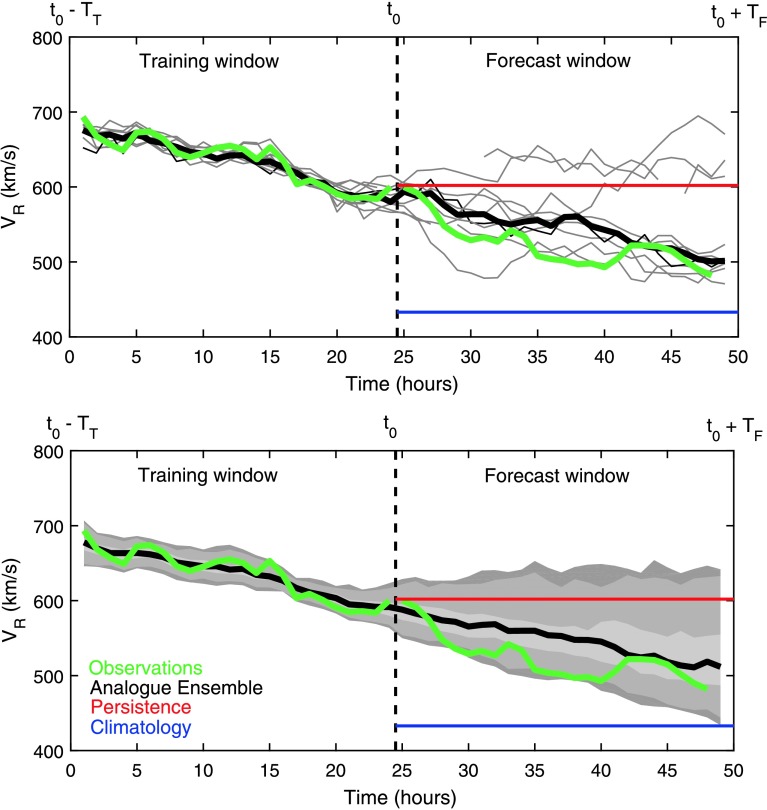
An example of an analogue ensemble (AnEn) for radial solar-wind speed [$V_{\mathrm{R}}$]. The forecast is made at $t_{0} = 0{:}00$ UT on 11 June 2003. The training window length [$T_{\mathrm{T}}$] and the forecast window length [$T_{\mathrm{F}}$] are both 24 hours. The solid green line shows the observed variation. The thin grey lines in the top panel show the ten closest analogues in the whole OMNI dataset to the observed variation in the training window. The thick black line shows the median of this ten-member ensemble. The grey shaded regions in the bottom panel show the bands containing 68%, 90%, and 95% of a 100-member ensemble. The red and blue lines show the persistence and climatological forecasts, respectively.

An example is shown in Figure 1 for the radial solar-wind speed [$V_{\mathrm{R}}$] variation on 11 June 2003. The training and forecast window lengths, $T_{\mathrm{T}}$ and $T_{\mathrm{F}}$, are both 24 hours. The green line shows the observed variation, which shows a fairly steady decline in both the training and forecast windows. The thin grey lines in the top panel show the ten closest analogues in the training window, drawn from the entire OMNI dataset. There is a great deal of spread in this ten-member ensemble during the forecast window, but the median, shown as the thick black line, does show a downward variation, as observed. This variation would not be obtained by assuming, *e.g.* persistence (shown in red) of the last observed value (approximately $600~\mbox{km}\,\mbox{s}^{-1}$) over the forecast window. Similarly, assuming the climatological value of $V_{\mathrm{R}}$, taken to be the mean over the whole OMNI dataset ($433~\mbox{km}\,\mbox{s}^{-1}$, shown in blue), would provide a poor forecast. The bottom panel shows the same analysis for $N_{\mathrm{EN}} =100$, with the grey shaded bands containing 67%, 90%, and 95% of the ensemble members. Such assessments of uncertainty are fully nonparametric and do not assume that the data are normally distributed.

Note that the top panel of Figure 1 shows a number of discontinuous lines, which are the result of data gaps in the forecast windows of individual analogues. As the forecast median and confidence intervals are calculated independently at each time step, data gaps are simply excluded. This means that when a large number of analogues contain data gaps at a given forecast time, the reliability of the median reduces and the width of the confidence intervals expands. We limit this effect by requiring at least 75% data coverage in both the training and forecast windows.

The quality of the forecast obtained in this manner depends critically on a number of parameters, particularly $T_{\mathrm{T}}$, $N_{\mathrm{EN}}$, and the choice of training parameter(s). In the initial study of Riley *et al.* (2017), $T_{\mathrm{T}}$ was fixed at 24 hours, $N_{\mathrm{EN}}$ was fixed at 50, and the closest analogues were determined only using the parameter being forecast. In order to assess the optimum values for solar-wind forecasting, we test a range of values and parameters for the period 1 January 2003 to 31 June 2003. While there is nothing particularly special about this period, it does contain some prolonged periods of fast and slow wind, as well as a number of interplanetary coronal mass ejections, testing the AnEn over a wide range of conditions. A longer interval is not used for computational reasons. It is not possible to fully explore the parameter space in a systematic fashion for the same reason. Instead, we explore each variable in turn and consider only the root-mean square error (RMSE) between observations and ensemble median. Note that by considering only the ensemble median, this method does not fully exploit the power of a probabilistic forecast, discussed further below. At 0:00 UT for each day in the six-month period, we compute the AnEn median forecast over the next 24 hours and compare that with observations (the analogue periods, however, can begin at any UT). Thus the RMSE is computed for a range of forecast lead times from 1 to 24 hours. Performance at each lead time is considered below.

**Figure 2:**
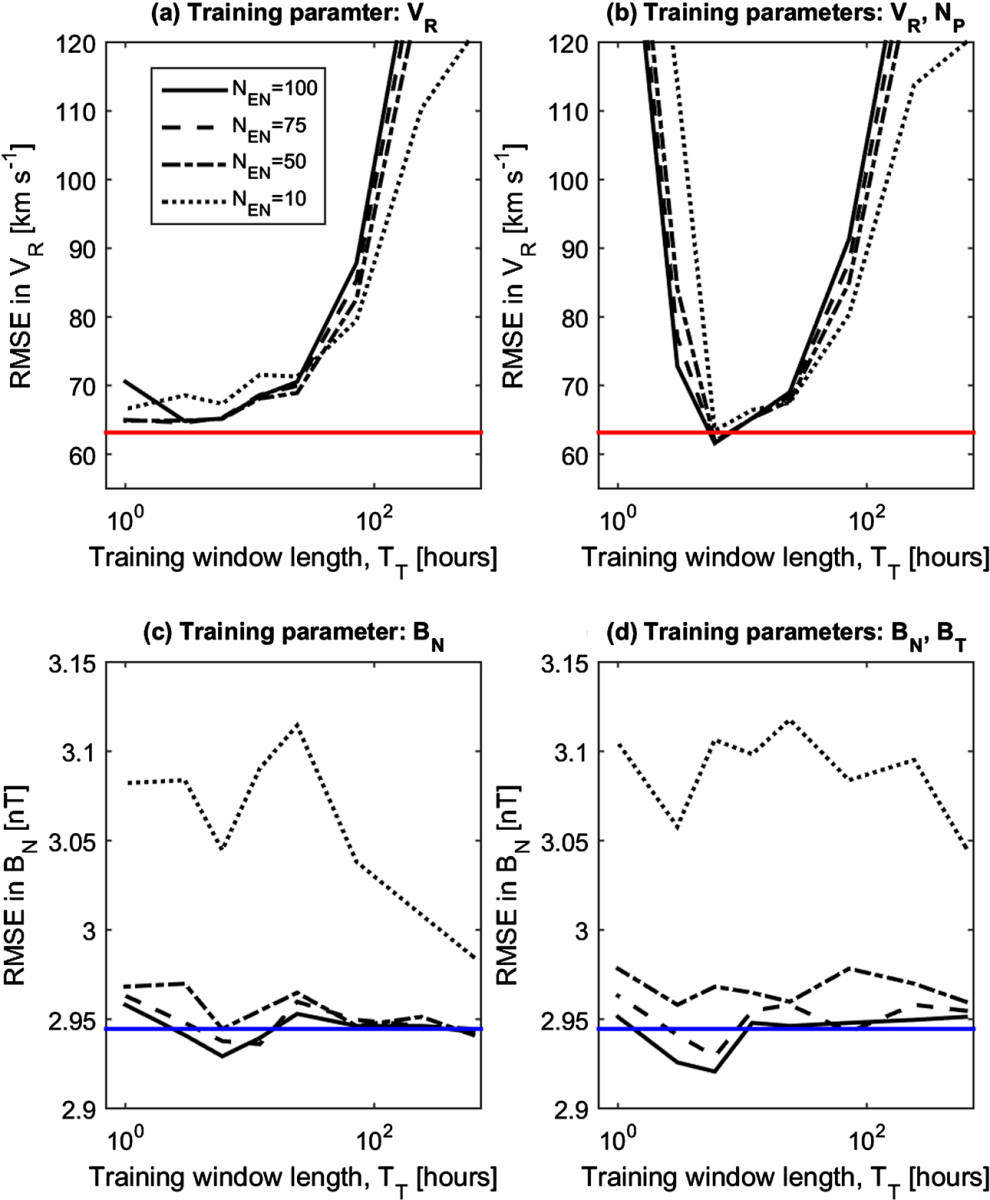
The RMSE between observations and the AnEn median over the period 1 January 2003 to 1 July 2003 for a range of training window lengths and number of ensemble members. Panels a and b show the RMSE in the median $V_{\mathrm{R}}$ forecast using analogues determined from (a) $V_{\mathrm{R}}$ only and (b) $V_{\mathrm{R}}$ and $N_{\mathrm{P}}$. Solid, dashed, dot–dashed, and dotted lines show $N_{\mathrm{EN}} =100$, 75, 50, and 10, respectively. The solid red lines show the best persistence forecast. Panels c and d show the RMSE in the $B_{\mathrm{N}}$ forecast using analogues determined from (c) $B_{\mathrm{N}}$ only and (d) $B_{\mathrm{N}}$ and $B_{\mathrm{T}}$. The solid blue lines show the climatological forecast.

Figure 2 shows the RMSE error in the AnEn median forecast over the six-month interval in early 2003 (although the training analogues are drawn from the whole OMNI dataset). Figure 2a is the $V_{\mathrm{R}}$ forecast using analogues determined using only $V_{\mathrm{R}}$ data. A range of training window lengths, from 1 hour to 27 days, and a range of number of ensemble members, from 10 to 100, are considered. For high values of $T_{\mathrm{T}}$ (> one day), the RMSE between observations and the AnEn median is generally very high. For high $T_{\mathrm{T}}$, RMSE also increases with $N_{\mathrm{EN}}$, as the number of suitable analogues becomes insufficient. A minimum RMSE of $64.8~\mbox{km}\,\mbox{s}^{-1}$ is found for $N_{\mathrm{EN}} =100$ and $T_{\mathrm{T}} = 6~\mbox{hours}$ (higher values of $N_{\mathrm{EN}}$ than shown in Figure 2, specifically, 150, 200, and 300, resulted in larger RMSE at all $T_{\mathrm{T}}$). See also Table 1. For comparison, the solid red lines in Figure 2a and b show the best persistence forecast for $V_{\mathrm{R}}$, namely that $V_{\mathrm{R}}$ over the next 24 hours is equal to the value at 0:00 UT. The high autocorrelation in $V_{\mathrm{R}}$ means that this is an extremely good forecast in terms of RMSE, at least over short lead times (*e.g.* averaged over forecast lead times from 1 to 24 hours, it outperforms all current numerical solar-wind models (Owens *et al.* 2008), as well as 27-day persistence (Owens *et al.* 2013)), although that does not necessarily make it a useful forecast for operational decision making. For this six-month test period, persistence results in an RMSE of $63.2~\mbox{km}\,\mbox{s}^{-1}$, lower than any of the AnEn forecasts considered in Figure 2a. The “best” AnEn parameters result in an RMSE of $64.8~\mbox{km}\,\mbox{s}^{-1}$, this suggest that at best the AnEn approaches persistence. Conversely, a climatological forecast of $V_{\mathrm{R}}$ (*i.e.* that $V_{\mathrm{R}} = 433~\mbox{km}\,\mbox{s}^{-1}$ at all times) is very poor, resulting in an RMSE of $190~\mbox{km}\,\mbox{s}^{-1}$ (hence it is not shown in the figure).

**Table 1:** Summary of the training parameters that give the lowest RMSE over the interval 1 January 2003 – 1 July 2003 for the AnEn median of various forecast parameters.

Forecast parameter	Best training parameter(s)	Best $N_{\mathrm{EN}}$	Best [hours]	$T_{\mathrm{T}}$ AnEn median	RMSE	
					Persistence	Climatology
$B_{\mathrm{T}}$	$B_{\mathrm{T}}$	100	6	$ 3.42~\mbox{nT} $	$ 4.19~\mbox{nT} $	$4.50~\mbox{nT}$
$B_{\mathrm{N}}$	$B_{\mathrm{T}}$, $B_{\mathrm{N}}$	100	6	$ 2.92~\mbox{nT} $	$ 4.24~\mbox{nT} $	$2.94~\mbox{nT}$
$V_{\mathrm{R}}$	$V_{\mathrm{R}}$, $N_{\mathrm{P}}$	100	6	$ 61.6~\mbox{km}\,\mbox{s}^{-1} $	$ 63.2~\mbox{km}\,\mbox{s}^{-1} $	$190~\mbox{km}\,\mbox{s}^{-1}$
$N_{\mathrm{P}}$	$V_{\mathrm{R}}$, $N_{\mathrm{P}}$	100	12	$ 2.79~\mbox{cm}^{-3} $	$ 4.22~\mbox{cm}^{-3} $	$3.49~\mbox{cm}^{-3}$

Of course, there is no reason why the analogue periods have to be selected solely on the basis of the forecast parameter (as in the $V_{\mathrm{R}}$ forecast discussed above). Figure 2b shows the RMSE in the AnEn median $V_{\mathrm{R}}$ forecast using analogue periods determined by both $V_{\mathrm{R}}$ and $N_{\mathrm{P}}$ in the training window. In order to combine RMSE in different solar-wind parameters measured in different physical units, it is necessary to first convert them into a normalised quantity. To achieve this, the cumulative distribution functions (CDFs) of $V_{\mathrm{R}}$ and $N_{\mathrm{P}}$ are computed over the whole OMNI dataset. The time series of $V_{\mathrm{R}}$ and $N_{\mathrm{P}}$ are then converted into rank within their respective CDFs. RMSE in the training window is then calculated on the basis of CDF rank. The $V_{\mathrm{R}}$ and $N_{\mathrm{P}}$ rank RMSEs are then multiplied together and the analogues are taken to be the periods with the lowest values.

Figure 2b shows that for $N_{\mathrm{EN}} >10$, the AnEn median forecast of $V_{\mathrm{R}}$ using analogues determined by both $V_{\mathrm{R}}$ and $N_{\mathrm{P}}$ performs better than persistence at $T_{\mathrm{T}} = 6~\mbox{hours}$. The lowest RMSE is for $N_{\mathrm{EN}} =100$ and $T_{\mathrm{T}} = 6~\mbox{hours}$, resulting in an RMSE of $61.6~\mbox{km}\,\mbox{s}^{-1}$. For the AnEn forecast of $N_{\mathrm{P}}$ (not shown), there is a similar situation: over the period January – June 2003, a persistence forecast of $N_{\mathrm{P}}$ gives an RMSE of $4.22~\mbox{cm}^{-3}$. A climatological forecast of $N_{\mathrm{P}} = 6.74~\mbox{cm}^{-3}$ gives an RMSE of $3.49~\mbox{cm}^{-3}$. The best AnEn median forecast for $N_{\mathrm{P}}$ trained on just $N_{\mathrm{P}}$ is $T_{\mathrm{T}} = 12$ hours and $N_{\mathrm{EN}} =100$, giving an RMSE of $2.88~\mbox{cm}^{-3}$. Training the AnEn on both $V_{\mathrm{R}}$ and $N_{\mathrm{P}}$, however, gives an $\mbox{RMSE} =2.79~\mbox{cm}^{-3}$ for $T_{\mathrm{T}} = 12$ hours and $N_{\mathrm{EN}} =100$.

Figure 2c shows the RMSE in $B_{\mathrm{N}}$ using analogues determined by $B_{\mathrm{N}}$. The dashed line shows the climatological forecast, *i.e.* $B_{\mathrm{N}} =0$, which results in an RMSE of 2.94 nT, better than the current numerical and 27-day persistence forecasts, although it is likely to be of little value as a forecast for operators. (The lack of autocorrelation in the $B_{\mathrm{N}}$ time series means that the 1- to 24-hour persistence forecasts of $B_{\mathrm{N}}$ are poor, giving an RMSE of 4.09 nT. Hence it is not shown in the figure.) For increasing $N_{\mathrm{EN}}$ and $T_{\mathrm{T}}$, the AnEn forecast tends towards climatology. For large $N_{\mathrm{EN}}$ (*i.e.* ${>}\,75$), the AnEn forecast using $T_{\mathrm{T}}$ between 3 and 12 hours has an RMSE that is just lower than persistence. Figure 2d shows the same results, but for analogues determined on the basis of both $B_{\mathrm{N}}$ and $B_{\mathrm{T}}$. For $N_{\mathrm{EN}} >50$ and $T_{\mathrm{T}}$ around 6 hours, the median AnEn forecast is improved slightly.

The optimum AnEn parameters and the resulting RMSE are summarised in Table 1. Obviously, it is also necessary to quantify whether the differences in RMSE between the various forecast types actually lead to a meaningful increase in an operator’s ability to successfully take action. The utility of a forecast depends greatly on the particular application. Thus in the probabilistic AnEn section, we also compute the “potential economic value” over a range of operational scenarios.

## Performance of the Deterministic Solar-Wind AnEn over 1996 – 2014

We now investigate the performance of the AnEn using the parameters in Table 1 over a much longer period, covering 1 January 1996 to 1 January 2015. This 19-year interval almost spans the period of near-complete OMNI data coverage. No attempt is made to remove or isolate the interplanetary manifestations of coronal mass ejections, which are treated in the exact same manner as any other solar-wind interval. Forecasts (be it AnEn, persistence, or climatological) are made at 0:00 UT for every day in this interval and the average RMSE computed for a range of lead times, from 1 hour to 30 days.

**Figure 3:**
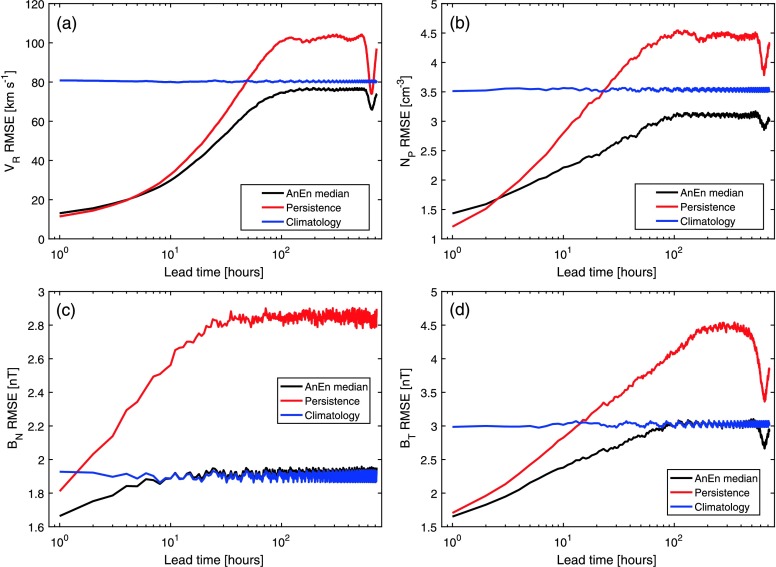
Average forecast RMSE over 1996 – 2015 as a function of forecast lead time for (a) $V_{\mathrm{R}}$, (b) $N_{\mathrm{P}}$, (c) $B_{\mathrm{N}}$, and (d) $B_{\mathrm{T}}$. Black, red, and blue lines show the AnEn, persistence, and climatological forecasts, respectively.

Figure 3 shows the average RMSE as a function of forecast lead time. Panels a, b, c, and d show forecasts of $V_{\mathrm{R}}$, $N_{\mathrm{P}}$, $B_{\mathrm{N}}$, and $B_{\mathrm{T}}$, respectively. The climatological forecasts (blue) assume that the mean values of the forecast parameters, computed over the entire OMNI dataset, persist at all times. Thus the average RMSE for climatology is approximately constant over all lead times, with a value of around $80~\mbox{km}\,\mbox{s}^{-1}$ for $V_{\mathrm{R}}$, $3.5~\mbox{cm}^{-3}$ for $N_{\mathrm{P}}$, 1.9 nT for $B_{\mathrm{N}}$, and 3 nT for $B_{\mathrm{T}}$. The persistence forecasts, in red, assume that the hourly values of the forecast parameters at 0:00 UT persist over the forecast window. For all parameters, the average RMSE for persistence is small initially (as the current value of, *e.g.*, $V_{\mathrm{R}}$ is closely correlated with the value one hour previously), but grows rapidly with increased forecast lead time. For $V_{\mathrm{R}}$, $N_{\mathrm{P}}$, and $B_{\mathrm{T}}$, persistence out-performs climatology for forecast lead times up to 15 – 30 hours. For $B_{\mathrm{N}}$, however, the RMSE for persistence is higher than climatology even at a lead time of two hours, demonstrating the short autocorrelation time in the $B_{\mathrm{N}}$ time series and the inherent difficulty in $B_{\mathrm{N}}$ prediction (*e.g.* Lockwood *et al.* 2016 and references therein).

The AnEn forecasts, in black, use the parameters outlined in Table 1. The RMSE is computed between the AnEn median and observed time series. For $V_{\mathrm{R}}$ and $N_{\mathrm{P}}$, persistence outperforms (*i.e.* provides a forecast with lower RMSE) the AnEn median for very short lead times, ${<}\,5$ and ${<}\,3$ hours, respectively. For longer lead times, the AnEn median is better than both persistence and climatology. The RMSE in the AnEn grows with increasing forecast lead time, but at a much slower rate than persistence. As for persistence, the AnEn RMSE plateaus at a forecast lead-time of around 100 hours, but the AnEn reaches a value lower than the climatological RMSE. This is due to the climatology being calculated over the whole OMNI dataset, which results in a different mean value than over the 1996 – 2015 test period, primarily as a result of solar-cycle sampling. The AnEn appears to be effectively selecting a more appropriate climatology. There is a small drop in the RMSE of the AnEn median and persistence forecasts of $V_{\mathrm{R}}$ and $N_{\mathrm{P}}$ at lead times of approximately 27 days (approximately 650 hours), which is due to the recurrence of solar-wind structures with solar rotation (*e.g.* Chree and Stagg 1928; Owens *et al.* 2013). For $B_{\mathrm{N}}$ and $B_{\mathrm{T}}$ forecasts, the AnEn median beats persistence and climatology for all lead times, although the AnEn median essentially regresses towards climatology for lead times of around 100 hours for $B_{\mathrm{T}}$ and 10 hours for $B_{\mathrm{N}}$. AnEn and persistence show the 27-day recurrence feature for forecasts of $B_{\mathrm{T}}$, but not $B_{\mathrm{N}}$.

## Testing the Probabilistic AnEn

Up to this point, only the AnEn median has been considered, essentially using the AnEn as a deterministic forecast. In order to assess the performance of the probabilistic AnEn forecast relative to the performance of deterministic forecasts such as persistence and climatology, we compute the potential economic benefit of the forecasts (Murphy 1977; Richardson 2000). This metric is best understood by an example (Owens *et al.* 2014): a spacecraft will suffer some kind of failure if a particular solar-wind parameter exceeds a threshold $X$. The expense of this failure is referred to as the loss [$L$]. Mitigating action, such as putting the spacecraft into safe mode, can be taken to protect the spacecraft, but this action also has a cost [$C$]. Thus, in the absence of a usable forecast, the spacecraft should always be in safe mode if the climatological probability of exceeding $X$ is greater than $C/L$. The total expense of operating the spacecraft is then simply $\mathit{NC}$, where $N$ is the number of time steps considered. If, on the other hand, the climatological probability of exceeding $X$ is lower than $C/L$, the spacecraft should operate continuously and the total expense will be $L$ multiplied by the sum of all of the times $X$ was actually observed to be exceeded. The expense can be similarly computed for acting on a deterministic forecast of $X$ being exceeded. For a probabilistic forecast, mitigating action should only be taken when the forecast probability of exceeding $X$ is forecast to be greater than $C/L$. *Potential economic value* (*PEV*) *compares the expense of acting on a given forecast with* both climatology and a perfect deterministic forecast (see Equation (1) of Owens *et al.* 2014), with 100 indicating a perfect forecast and values below 0 indicating the forecast is less effective than climatology.

**Figure 4:**
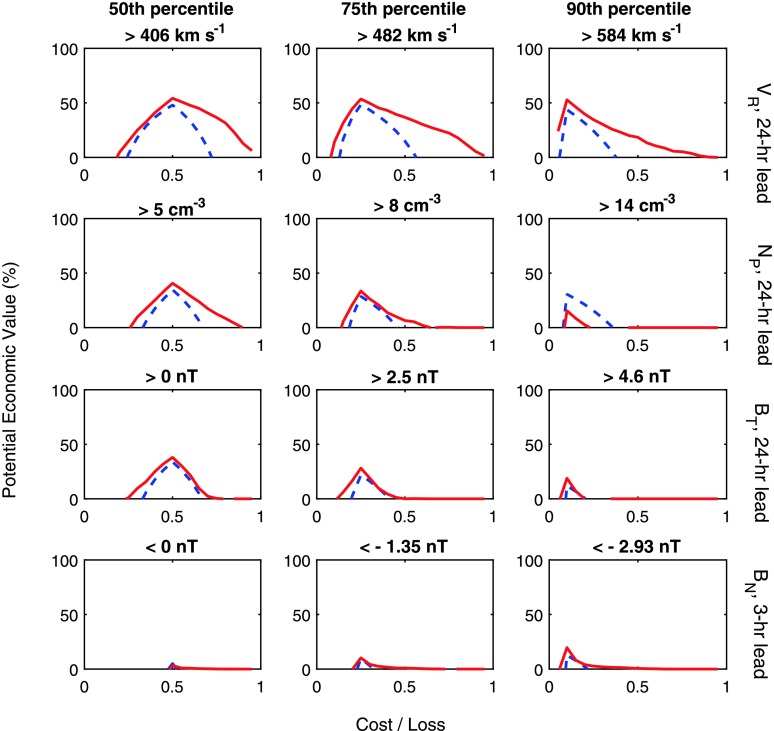
The potential economic value of a forecast (relative to climatology) for a range of cost/loss ratios, where cost is the expense of taking mitigating action and loss is the expense of not taking action during adverse space-weather conditions. Red lines show the AnEn, blue dashed lines show persistence. The rows show from top to bottom forecasts for $V_{\mathrm{R}}$, $N_{\mathrm{P}}$, and $B_{\mathrm{T}}$ with a 24-hour lead time, and $B_{\mathrm{N}}$ with a 3-hour lead time. The columns show from left to right an increasing threshold for taking action, namely the 50th, 75th, and 90th percentile of the forecast parameter.

Figure 4 shows the PEV of persistence (blue dashed) and AnEn (red) forecasts for a range of solar-wind parameter thresholds and cost/loss ratios. PEV is computed over the whole 19-year interval. The rows show from top to bottom $V_{\mathrm{R}}$, $N_{\mathrm{P}}$, and $B_{\mathrm{T}}$ forecasts with a 24-hour lead time, and $B_{\mathrm{N}}$ forecasts with a 3-hour lead time ($B_{\mathrm{N}}$ with a 24-hour lead time has $\mbox{PEV} <0$ for all cost/loss ratios, thresholds and across the AnEn and persistence, and so it is not shown). The columns show from left to right increasing thresholds for mitigating action, namely the 50th, 75th, and 90th percentiles of the forecast parameters. For $V_{\mathrm{R}}$, this means thresholds of 408, 482, and $584~\mbox{km}\,\mbox{s}^{-1}$. For $B_{\mathrm{N}}$, negative percentiles and thresholds are considered, *i.e.* below 0, −1.35, and $-2.93~\mbox{nT}$.

It can be seen that the AnEn $V_{\mathrm{R}}$ forecasts are “valuable” (in providing a more actionable forecast than climatology and thus having a potential economic value greater than zero) at nearly all cost/loss ratios and mitigation thresholds. The AnEn value is also greater than persistence at all cost/loss ratios and thresholds. For $N_{\mathrm{P}}$, the AnEn provides a more valuable forecast than persistence for low and medium thresholds, but a poorer forecast for high thresholds. The 24-hour lead time AnEn forecast of BT provides an improvement over persistence over most cost/loss ratios, although this advantage decreases as the threshold for action is increased. For $B_{\mathrm{N}}$, even at 3-hour lead times, the value of the forecasts is small. The AnEn forecast, however, does show a value greater than climatology and persistence over a wide range of cost/loss ratios for moderate and large negative $B_{\mathrm{N}}$ excursions.

**Figure 5:**
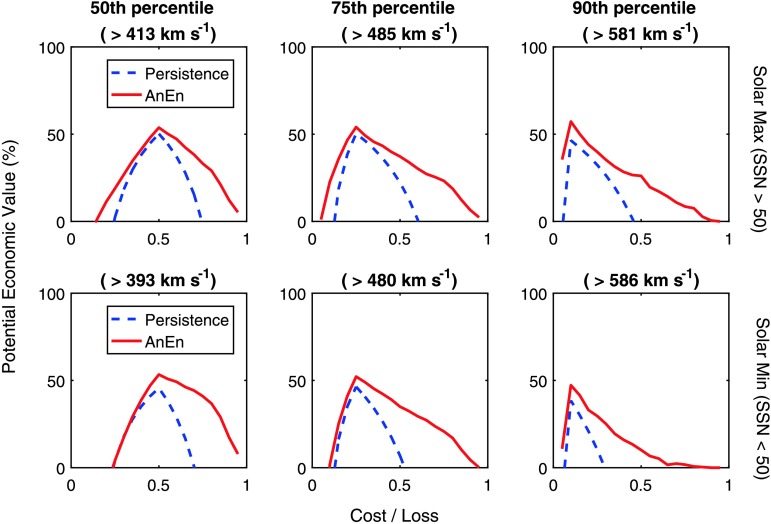
The potential economic value of solar-wind speed forecasts (relative to climatology) for a range of cost/loss ratios, where cost is the expense of taking mitigating action and loss is the expense of not taking action during adverse space-weather conditions. In the same format as Figure 4. The top panels show solar-maximum periods, the bottom panels solar-minimum periods. The rows show from left to right action thresholds at the 50th, 75th, and 90th percentiles of the solar-maximum and -minimum periods.

A full investigation of the performance of the AnEn over the solar cycle would require tuning the AnEn parameters (*e.g.* $N_{\mathrm{EN}}$ and $T_{\mathrm{T}}$) at each point of the solar cycle. As a preliminary investigation, here we simply use the optimum values determined over the whole dataset (as listed in Table 1) to assess the performance in solar maximum and solar minimum, distinguished using a monthly smoothed sunspot-number threshold of 50, which approximately bisects the dataset. Results for the solar-wind speed [$V_{\mathrm{R}}$] are shown in Figure 5. The performance of the AnEn (and persistence) is broadly similar between the two periods, but, somewhat counterintuitively, the AnEn appears to perform marginally better during solar maximum. This may be the result of reduced $V_{\mathrm{R}}$ variability at this time (note the reduced range of the percentiles in solar maximum compared with solar minimum).

## Geomagnetic Forecasting

We now apply the AnEn methodology to forecasting of geomagnetic parameters. We consider the two most widely used geomagnetic indices: Dst and Kp. Dst is obtained from equatorial magnetometers and is therefore primarily a measure of the ring-current intensity, with high negative values that are indicative of an on-going geomagnetic storm (Sugiura 1964). Kp is a measure of the range of magnetic variability observed by mid-latitude stations, scaled between 0 and 9, with larger numbers indicating magnetically disturbed periods (Bartels, Heck, and Johnston 1939).

**Figure 6:**
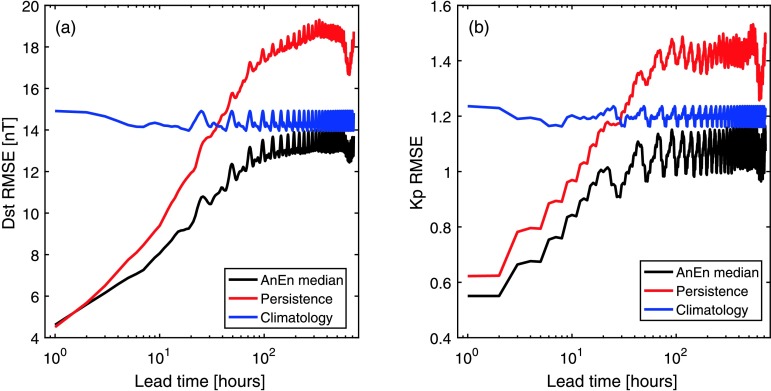
Average forecast RMSE over 1996 – 2015 as a function of forecast lead time for (a) Kp and (b) Dst. Black, red, and blue lines show the AnEn, persistence, and climatological forecasts, respectively.

Figure 6 shows the average forecast RMSE in Dst and Kp over 1996 – 2015 as a function of forecast lead time. As for Figure 3, the AnEn median is shown in black, persistence in red, and climatology in blue. For both AnEn forecasts, the training window is 6 hours and the number of ensemble members is 100. The analogues are determined purely from the forecast parameters (*i.e.* just Dst for the Dst forecast and just Kp for the Kp forecast). When we use the current AnEn methodology, the inclusion of $B_{\mathrm{N}}$ and/or $V_{\mathrm{R}}$ as training parameters is found to produce little to no improvement in the forecast RMSE.

All forecasts show a strong diurnal variation in RMSE, resulting from the tilt of the Earth’s rotational axis relative to the Ecliptic plane, which leads to a diurnal variation in the coupling efficiency between the heliospheric and magnetospheric magnetic fields (Siscoe and Crooker 1996). Consequently, the best forecast (*i.e.* lowest RMSE) within a given 24-hour range of lead times occurs at the same time of day as the time the forecast is made (*i.e.* 0:00 UT in this case). For both Dst and Kp, persistence provides a better forecast than climatology out to around 24 hours lead time. The AnEn median provides a lower RMSE at all lead times beyond one hour and continues to provide a useful forecast out to around four days. In fact, the ability of the AnEn to select an appropriate climatology means it provides a better forecast than a simple climatological means for all lead times. Both persistence and the AnEn median show a decrease in forecast RMSE for forecast lead times of around 27 days, as expected.

**Figure 7:**
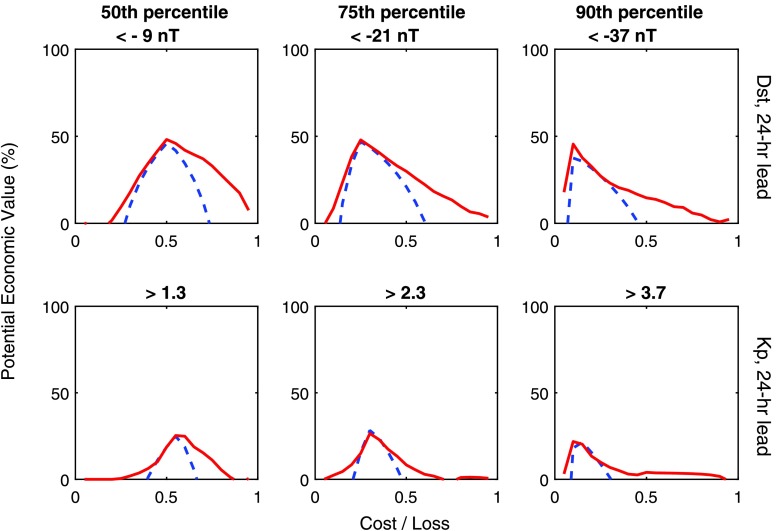
The potential economic value of a forecast (relative to climatology) for a range of cost/loss ratios, where cost is the expense of taking mitigating action and loss is the expense of not taking action during adverse space-weather conditions. Red lines show the AnEn, blue dashed lines show persistence. The top row shows the Dst forecast, the bottom row the Kp forecast. The columns show from left to right an increasing threshold for taking action, namely the 50th, 75th, and 90th percentile of the forecast parameter.

The performance of the probabilistic AnEn is assessed in Figure 7. The potential economic value of acting on the 24-hour lead-time AnEn and persistence forecasts of Dst and Kp are shown in the top and bottom rows, respectively. The AnEn forecast is comparable to, or better than, persistence for all thresholds and cost/loss ratios for both Dst and Kp, demonstrating the value of this approach for forecasting geomagnetic disturbances. The level of improvement provided by the AnEn over persistence, however, is a strong function of the cost/loss ratio, which is fixed by the application. So the benefit provided by the use of the AnEn will be strongly application dependent. These results also confirm that the AnEn provides a useful measure of the forecast uncertainty, as well as the most probable value. We also note that relative to climatology, Dst appears to be far more predictable via both the AnEn and persistence than Kp.

## Summary and Conclusions

We have investigated the use of an analogue ensemble (AnEn), often referred to as a “similar day” approach, for probabilistic solar-wind and geomagnetic forecasting. This forecast is constructed by determining a number of periods in the historical data that are “analogous” to the current conditions. It is then assumed that future variations will follow the same trends as these analogues. If an ensemble of analogues is used, the forecast is inherently probabilistic. As outlined by Riley *et al.* (2017), the AnEn approach is very promising for short or medium lead-time solar-wind and geomagnetic forecasting (hours to days) and thus may serve as a complementary approach to the longer lead-time (days to weeks) physics-based magnetohydrodynamic models.

Forecasts for four solar-wind parameters were considered, chosen for their geomagnetic relevance. They are the solar-wind radial speed [$V_{\mathrm{R}}$], the solar-wind density [$N_{\mathrm{P}}$], the meridional magnetic-field component in heliographic coordinates [$B_{\mathrm{N}}$] (which is approximately the southward magnetic field [$B_{\mathrm{Z}}$] in geocentric-solar-ecliptic, GSE, coordinates), and the tangential magnetic field [$B_{\mathrm{T}}$] (which is approximately $B_{\mathrm{Y}}$ in GSE coordinates).

A six-month interval of solar-wind data from the first half of 2003, including both ambient solar wind and transient structures from coronal mass ejections, was used to determine the optimum AnEn parameters. One of the most critical parameters is the length of the “training window” over which historical analogues are compared to current solar-wind conditions. In general, we found that around 6 – 12 hours was the optimum value in terms of minimising the root mean-square error (RMSE) of the AnEn median. Increasing the length of the training window beyond around 24 hours generally increased the RMSE of the resulting forecast, as it reduces the number of suitable analogues in the historical dataset. As the suitability of analogues decreases, the ensemble median forecast essentially reduces to climatology. A longer training window also decreases the relative weighting of the most recent observations, which may produce less suitable analogues for future variations. The AnEn RMSE is also found to increase if the number of analogues in the ensemble is increased above around 100, as insufficient analogues are present in the currently available historical dataset (around 60 years of near-Earth spacecraft observations). For solar-wind forecasting, the determination of the analogue periods was found to benefit from additional contextual information. For example, the AnEn $V_{\mathrm{R}}$ forecast is improved when both $V_{\mathrm{R}}$ and $N_{\mathrm{P}}$ data are considered in the training window, while the $B_{\mathrm{N}}$ forecast improves when both $B_{\mathrm{N}}$ and $B_{\mathrm{T}}$ are considered. There is a trade-off, however, with increased specificity meaning that the number of suitable past analogues decreases and emphasis on the forecast parameter is reduced.

Using the optimal parameters, the AnEn forecasts were subsequently tested over a 19-year period of nearly complete solar-wind observational coverage, 1996 – 2015. As most common space-weather metrics necessitate a deterministic forecast, it is instructive to first reduce the AnEn to a deterministic forecast by considering only the median value. At very short forecast lead times (one to six hours), the high autocorrelation in the solar-wind plasma parameters means that a persistence forecast of $V_{\mathrm{R}}$ and $N_{\mathrm{P}}$ is more accurate than the AnEn median or climatology. That advantage is rapidly lost with increasing lead time, with the AnEn median providing a much lower RMSE than persistence for lead times longer than a few hours. With increasing lead time, the RMSE of the AnEn grows, although at a slower rate than persistence. For lead times longer than around 100 hours (approximately four days), the RMSE of the AnEn forecast of $V_{\mathrm{R}}$ and $N_{\mathrm{P}}$ plateaus, but to a value below climatology. This is due to the AnEn effectively selecting a more suitable “climatology” for the forecast window than is given by simply averaging over the whole dataset, which can skew the mean towards, *e.g.*, the wrong phase of the solar cycle.

Using the cost/loss method of determining the effectiveness of a probabilistic forecast, the 24-hour lead time AnEn forecast was shown to generally outperform persistence. For $B_{\mathrm{N}}$, however, there is little value in the AnEn (or persistence) 24-hour lead-time forecasts relative to climatology (*i.e.* $B_{\mathrm{N}} = 0$). When the lead time is reduced to three hours, however, the AnEn forecast has value, particularly for the extreme negative values that are of prime interest to space-weather forecasting.

Finally, we considered the application of the AnEn to geomagnetic forecasting. The two most commonly used geomagnetic indices, Dst and Kp, were considered. The AnEn approach proved better than persistence for both indices over all forecast lead times and all cost/loss ratios.

## Future Improvements

The solar-wind AnEn forecasts outlined in this study are by no means the best possible AnEn forecasts. Indeed, there should not be considered to be a single solar-wind AnEn, as forecasts should be optimised for the required operational use. Some may emphasise a particular forecast lead time, some a more accurate “best” prediction, some a more accurate assessment of the forecast uncertainty, *etc*. With this in mind, we outline a number of possible future improvements to solar-wind AnEn forecasting:

- A more systematic exploration of the AnEn parameter space is required, ideally using a longer training dataset than the six months considered here. The limiting factor is computation time.
- The AnEn parameterisation should be investigated using the cost/loss analysis, rather than just the RMSE of the AnEn median, in order to find the best probabilistic forecast. The potential issue is reducing the multi-dimensionality of the problem.
- Preliminary results suggest that stratifying the dataset by solar-cycle phase may help in distinguishing between solar-wind structures expected in the given forecast window, such as corotating interaction regions in the declining phase of the solar-activity cycle.
- Only basic solar-wind parameters (plasma and magnetic field) were considered for determining the analogue periods, but other datasets may be able to give better contextual information. *E.g.* solar-wind compositional and charge-state data (*e.g.* Geiss, Gloeckler, and von Steiger 1995; Lepri and Zurbuchen 2004) may be useful for determining the solar-wind types being encountered.
- Longer training windows (days to weeks) may be feasible and useful if the analogue periods are weighted towards the most recent observations.
- Similarly, it may be helpful to use a greater number of solar-wind parameters in determining the analogue periods if these training parameters can be accurately weighted towards the information that they contain for future variations of the forecast parameter (*e.g.* when forecasting $V_{\mathrm{R}}$, it may be useful to determine the analogue periods on the basis of $V_{\mathrm{R}}$ with a high weighting, $N_{\mathrm{P}}$ with a medium weighting, and $T_{\mathrm{P}}$ with a low weighting).
- In this study, past analogues were selected by minimising RMSE with the recent observations. A more sophisticated pattern-matching or machine-learning algorithm may provide a better AnEn forecast. This would essentially combine the neural network and AnEn approaches.
